# Cortical representation of auricular muscles in humans: A robot-controlled TMS mapping and fMRI study

**DOI:** 10.1371/journal.pone.0201277

**Published:** 2018-07-27

**Authors:** Jonna Meincke, Manuel Hewitt, Markus Reischl, Rüdiger Rupp, Carsten Schmidt-Samoa, David Liebetanz

**Affiliations:** 1 Clinic of Clinical Neurophysiology, Georg August University of Göttingen, University Medical Center, Göttingen, Germany; 2 Institute for Applied Computer Science, Karlsruhe Institute of Technology, Eggenstein-Leopoldshafen, Germany; 3 Spinal Cord Injury Center, Heidelberg University Hospital, Heidelberg, Germany; 4 Department of Cognitive Neurology, Georg August University of Göttingen, University Medical Center, Göttingen, Germany; Universita degli Studi di Verona, ITALY

## Abstract

**Background:**

Most humans have the ability to activate the auricular muscles. Although (intentional) control suggests an involvement of higher cortical centers underlying posterior auricular muscle (PAM) activation, the cortical representation of the auricular muscles is still unknown.

**Methods:**

With the purpose of identifying a possible cortical representation area we performed automated robotic and image-guided transcranial magnetic stimulation (TMS) mapping (n = 8) and functional magnetic resonance imaging (fMRI) (n = 13). For topographical comparison, a similar experimental protocol was applied for the first dorsal interosseus muscle (FDI) of the hand.

**Results:**

The calculated centers of gravity (COGs) of both muscles were located on the precentral gyrus with the PAM COGs located more laterally compared to the FDI. The distance between the mean PAM and mean FDI COG was 26.3 mm. The TMS mapping results were confirmed by fMRI, which showed a dominance of cortical activation within the precentral gyrus during the corresponding motor tasks. The correspondence of TMS and fMRI results was high.

**Conclusion:**

The involvement of the primary motor cortex in PAM activation might point to an evolved function of the auricular muscles in humans and/or the ability of intentional (and selective) muscle activation.

## Introduction

**Fig 1:**
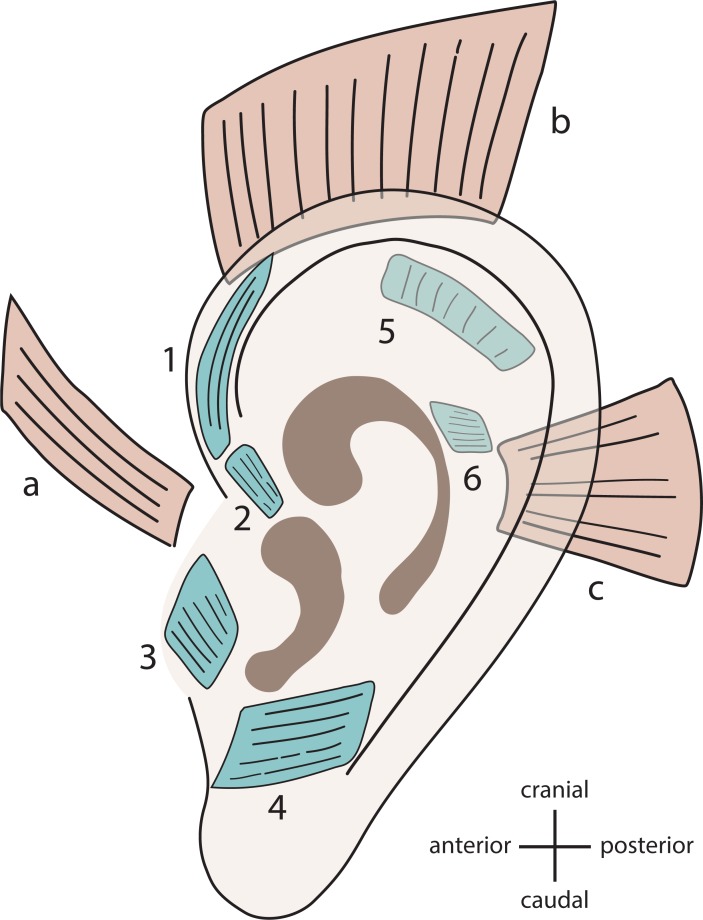
Human auricular muscles. Extrinsic (a—c) and intrinsic (1–6) auricular muscles. a = anterior auricular muscle, b = superior auricular muscle, c = posterior auricular muscle; 1 = helicis major, 2 = helicis minor, 3 = tragicus, 4 = antitragicus, 5 = oblique, 6 = transverse; the 5 and 6 are located on the back of the pinna.

The auricular muscles in humans consist of two subsets of intrinsic and extrinsic muscles with a total of nine muscles per ear (Fig 1). The intrinsic muscles both originate and insert at the auricular cartilage. The extrinsic auricular muscles have their origin at the cranial bone. Their contraction evokes motion of the pinna [1–3]. Besides its integration in short-latency brainstem reflexes [4, 5] the exact function in humans has not been identified so far and they are expected to be rudimentary muscles [6]. This is in contrast to non-human species where the auricular muscles play an important role in locating acoustical sources. Charles Darwin proposed that this function might have disappeared in some species during domestication [7].

Although the auricular muscles are probably rudimentary, it has been discussed to use the auricular muscles to control assistive devices [3, 8]. In a recent report EMG signals from the posterior auricular muscle (PAM) were used as a control signal for wheelchair control for tetraplegic patients [8]. Compared to classical control interfaces for tetraplegics (e. g. chin control), a control system based on auricular muscle activation has the advantage of minimal interference with speaking or laughing for example. The concept of using PAM activation as a control signal for electrical wheelchairs includes that these rudimentary muscles would obtain an entire new motor function. In this context, their neuroanatomy is of special interest. Tract-tracing studies in in non-human primates suggest that cortical control of the auricular muscles is mainly executed by supplementary and cingulate cortices [9, 10] but, in contrast to facial muscles the neuroanatomy of auricular muscle activation in humans has not been studied to date. However, intentional control and the ability to improve muscle activation indeed point to an involvement of higher cortical centers, such as the primary motor cortex, in auricular muscle activation.

With the purpose of identifying and locating a possible cortical representation of the auricular muscles we performed a transcranial magnetic stimulation (TMS) mapping study, which is a commonly used method to identify cortical representation areas of different muscles noninvasively [11–13]. It consists of delivering TMS pulses at different scalp positions in order to identify the optimal coil position for eliciting MEPs in the respective muscle. As inaccuracies of coil positioning, especially the tilt angle,strongly influence the resulting MEPs an exact and reliable coil positioning is crucial for TMS experiments [14–18]. Therefore, instead of manual coil positioning we used a novel automated image-guided and robot-controlled TMS system that ensured an exact coil positioning with respect of the six degrees of freedom to exclude any influence of the investigator and inaccurate coil positioning on the results [12]. This robotic system further enabled to apply the pulses in an entirely randomized sequence from pulse to pulse and over all targets. The influence of different states of alertness of the subjects during the experiments was therefore minimized [19–21] and the influence of the variability of MEPs reduced by applying a total of 12 pulses per target [21]. To confirm the TMS mapping results in a cross-modal approach we also performed functional magnetic resonance imaging (fMRI) during an auricular muscle motor task and compared the results of both methods. For neuroanatomical comparison a similar experimental protocol (TMS and fMRI) was performed for a hand muscle.

## Materials and methods

A total of 13 healthy subjects, 10 males, age 21–38 years (mean: 25.7), participated in the study. All were right-handed according to the Edinburgh handedness inventory [22]. All subjects gave their written informed consent for the study. 8 subjects participated in both TMS and fMRI mapping experiments, the remaining 5 subjects only in the fMRI experiments. The study protocol was approved by the ethics committee of the University of Göttingen and complied with the Declaration of Helsinki.

## Summary of the experiment

In the first step anatomical MRI data was acquired in 8 subjects. These subjects participated in two TMS mapping sessions for the FDI and for the PAM respectively. For each muscle and subject, the first mapping session served to define the stimulation parameters for the second (final) mapping session. This two-staged mapping procedure enabled standardized experiments but adapted to inter-individual differences (e. g. stimulation threshold).

The first mapping session was for the FDI muscle with 6 pulses per grid point. After the first session RMT was determined at the COG. The second session was performed on another day with the grid centered at the COG of the first session. The stimulation intensity for the second session was adjusted to 120% of the RMT from the first session.

The next step of the experiment was performed on another day. Prior to the mapping session a control experiment was performed to exclude that the TMS noise artifact would trigger the posterior auricular muscle reflex (PAMR). Then subjects were tested for the ability to generate EMG signals in the PAM on request. Subjects who could generate EMG signals >100μV directly participated in the PAM mapping session (see below). Subjects who failed to generate EMG signals >100μV participated in an extra EMG session and learned how to activate the PAM using EMG feedback (3 subjects). In this case the PAM TMS mapping session took place on another day.

The TMS mapping of the PAM included 2 mapping sessions. For the first TMS mapping of the PAM the grid was centered 2 cm anterior and lateral to the FDI COG. 6 pulses were applied per target and stimulation intensity was 140% of FDI RMT. After the first session a recruitment curve was measured at the COG. The second session was performed on another day. The stimulation intensity was set to the intensity at the inflection point of the recruitment curve and the grid was centered at the COG of the first PAM mapping session. The number of pulses was increased to 12 per grid point. After the second mapping session a final recruitment curve was measured at the COG.

The 8 subjects from the TMS experiment and 5 additional subjects participated in an additional fMRI mapping. The fMRI and TMS experiments were performed and analyzed independently.

### TMS of the posterior auricular muscle and first dorsal interosseus muscle

8 subjects participated in 4 TMS mapping sessions. TMS mapping was performed for the right PAM and the right first dorsal interosseus muscle (FDI). The subjects wore earplugs and were seated on a reclining chair during the TMS experiments. For TMS we used a Magstim 200^2^ magnetic pulse stimulator and a figure-of-eight coil (70 mm, Magstim Company, Whitland, UK) with a peak magnetic field of 2.2 T at the maximum stimulator output intensity (MSO). The coil was placed tangentially on the scalp and rotated 45 degrees to the sagittal plane. The coil touched the scalp but did not push the subject’s head. The exact positioning of the TMS coil was performed by an image-guided robotic system (see below). The interstimulus interval varied between 5 and 8 seconds, depending on the different durations of the robotic arm’s movements. The minimum interval between two TMS pulses was set to 5 seconds.

Prior to the PAM mapping session, each subject was tested for his/her ability to activate PAM on request. Subjects were asked to perform a backward movement of the pinna as strongly as possible. The EMG signal was presented on a computer screen in addition to an acoustic feedback. Those subjects, who were not able to generate PAM EMG signals of at least 100 μV on request, learned in an extra EMG session on how to activate the PAM (3 subjects). Here the PAM EMG signal was presented on a computer screen (biofeedback) and subjects trained how to activate the PAM >100 μV on request.

Prior to the mapping session we tested all subjects in a control experiment if the TMS noise artifact would trigger the PAMR [4]. The PAMR is a bilateral sound-evoked muscle activation of the PAM, which can be recorded in most subjects about 10 ms after an acoustical stimulus [4, 5, 23]. To exclude the PAMR, a control experiment was performed. The coil was placed vertically on the subject’s head (center of the grid of the first PAM mapping experiment) i. e. presenting the noise artifact without stimulating the cortex and a recruitment curve was measured with 10 stimuli per intensity.

Fine wire electrodes (diameter 50 μm) were used for EMG recording from PAM to increase signal quality. Using fine wire electrodes also minimized crosstalk from distant muscles and avoided a possible contamination of MEPs by blink reflex, which has the same latency and same shape as MEPs. The blink reflex is triggered by a direct stimulation of the afferent trigeminal nerve [24] and consists of a reflex activation of the orbicularis oculi muscle. However, in case of PAM a contamination of the MEPs by the blink reflex due to volume conduction is unlikely, as this muscle is located behind the pinna and relatively distant from the further facial muscles.

**Fig 2:**
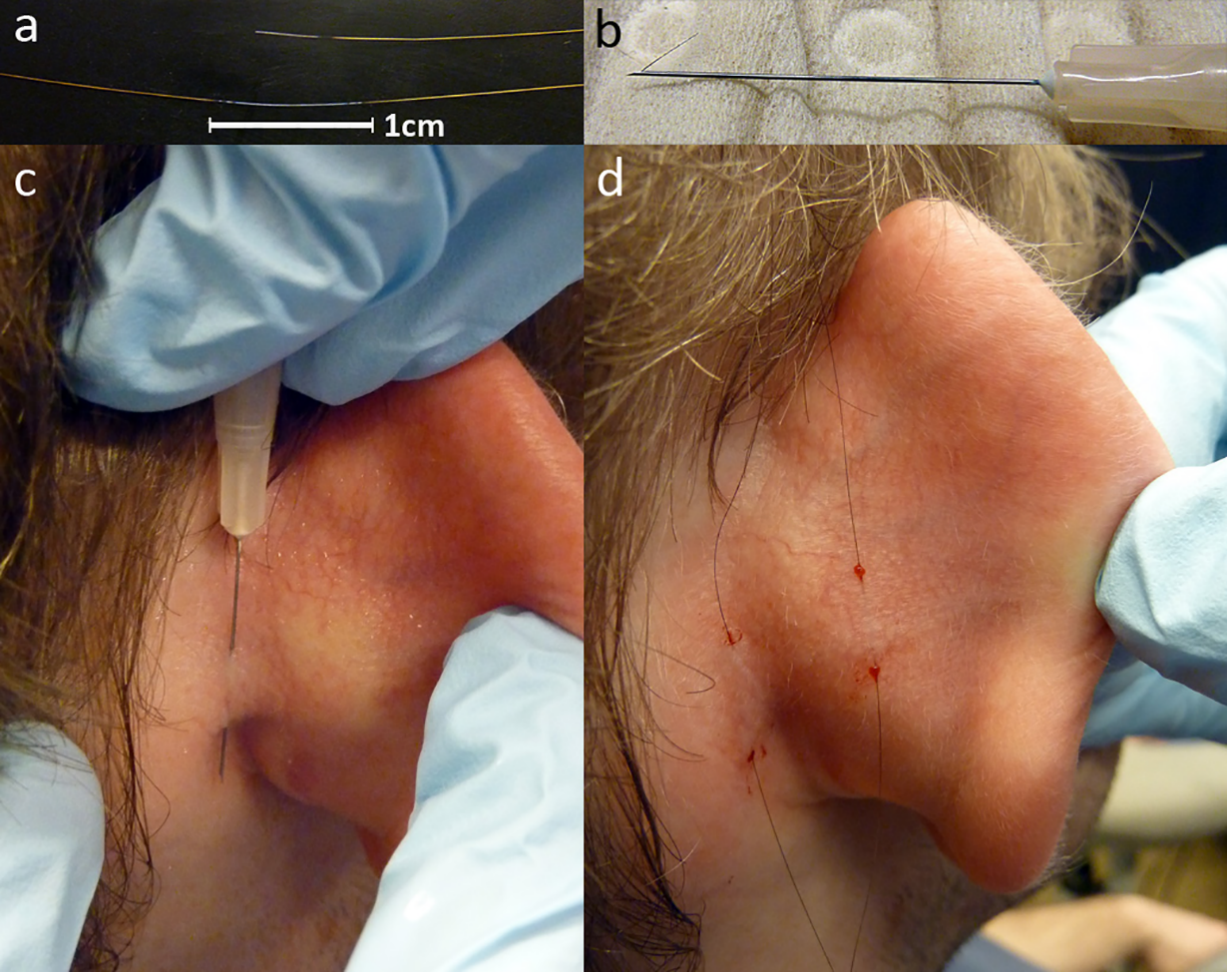
Placement of fine-wire electrodes. Fine-wire electrode with a total length of about 10 cm and a diameter of 50μm. Insulation was removed at both ends and along 1cm in the middle of the electrode (a). The electrode positioned within the injection needle (b). After piercing the skin twice, the electrode is retained and the needle is pulled back. The position of the electrode can be easily adjusted by pulling at either side. The stripped middle part of the electrode is located under the skin (c). Both electrodes in situ with the active electrode within the PAM (left) and the reference electrode in the middle of the pinna (right) (d).

For EMG recordings the active electrode was placed into PAM, and the reference electrode subcutaneously in the middle of the pinna (Fig 2). This electrode placement has been previously identified as being most effective for EMG recordings in the PAM (O´Beirne and Patuzzi, 1999). TMS of the PAM was performed during pre-innervation. Subjects were cued to activate the PAM by an acoustic signal that was presented 1 second prior to the TMS pulse. Pre-innervation was controlled on-line during a 200 ms interval prior to the stimulus. Sweeps in which a subject failed to pre-activate the PAM were automatically rejected and repeated later. Motor evoked potentials (MEPs) from the FDI were recorded with the muscle at rest using surface electrodes (Ag-AgCl) in a belly-tendon montage.

The signal was amplified and band-pass filtered from 2–2000 Hz (Digitimer D360, Digitimer Ltd.), sampled at 5000 Hz by an A/D converter (micro CED 1401 mkII, Cambridge Electronic Design) and recorded by software (Signal v4, CED) on a standard PC.

### Robot-controlled and image-guided TMS mapping procedure

We used a robot-controlled and image-guided TMS mapping technology to increase the accuracy and reliability of TMS [12, 25]. In this study, image-guided TMS mapping was further improved by a robot-controlled, high-precision coil positioning [12]. This procedure ensured a high accuracy and constant control of the coil position with respect to the 6 degrees of freedom and a high level of inter-session reliability [12, 25]. To further reduce the influence of MEP variability on the results [21] a relatively high number of pulses were applied at each target. Additionally, the automated coil positioning enables a randomized sequence of the stimulated targets, so that confounders such as fluctuations of cortical excitability of the subject [20] were balanced out over the grid and should not have biased the mapping results.

Prior to the experiments 3D head models were generated from cranial MRI data of each participant (T1 weighted, scanning parameters see below) by a special robot-navigation software (ANT, Enschede, Netherlands). At the beginning of each mapping session the shape and position of the subject’s head were registered to the head model. For this registration an optical tracking system, consisting of an infrared tracking camera and reflector lenses worn by the subjects, was used (NDI Polaris Vicra). The tracking system also compensated for head movements during the experiments. The positional error of the tracking system is ± 0.5 mm (specified by NDI). For the TMS mapping the robot navigation software created the target grid. An Adept Viper s850 robotic arm (Adept Technology, Inc. Livermore, CA, USA) then automatically and precisely positioned the TMS coil at the respective targets (positional error: ± 0.02 mm, specified by Adept) in a randomized order.

A two-staged mapping procedure was performed for each muscle, starting with the FDI. The grid consisted of 49 grid points (7x7 with 10 mm spacing). To locate the center of the grid and to adjust the intensity of stimulator output for the first mapping experiment, the best stimulation site to elicit MEPs of 0.5–1 mV in the FDI was located manually. Then a provisional resting motor threshold (RMT) was determined using an automated maximum-likelihood threshold-hunting algorithm [26]. The first TMS mapping experiment consisted of a total of 6 TMS-pulses over each grid point. The sequence was randomized. The stimulation intensity was set at 120% of the provisional RMT. The center of the grid was positioned at the manually determined best stimulation site. Sweeps in which subjects failed to relax the muscle were automatically rejected and repeated later.

Based on the data of the first TMS mapping session the center of gravity (COG) was calculated and an updated RMT was determined at the COG. In the following TMS session, which was performed on another day, the grid center was adjusted to the COG. This procedure ensured that the cortical representation area and the COG were located in the grid center of the final mapping experiment. The stimulation intensity was set at 120% of the updated RMT and the number of pulses per grid point was increased to 12. At the end, a decisive COG was calculated and the final RMT determined at the decisive COG.

The first PAM mapping session was performed on another day. For PAM mapping, the grid center was moved 2 cm both in an anterior and lateral direction and the intensity of stimulator output was set to 140% of the final FDI RMT. The first mapping session of the PAM consisted of 6 TMS pulses per target. Pulses were applied in a randomized order. Sweeps in which subjects failed to pre-activate the PAM (EMG signals >100μV) were rejected and repeated later. An initial COG was calculated based on this data. As PAM mapping was performed during pre-innervation, an RMT could not be acquired. Therefore, a recruitment curve with randomized intensities of stimulator output (between 10 and 100%, steps of 10%, 12 pulses per intensity) was recorded to determine the intensity of stimulator output for the following mapping experiment. One subject did not tolerate 90 and 100% intensity of stimulator output. In this case the recruitment curve was from 10 to 80%. For the second PAM mapping session, which took part on another day, the grid was centered on the initial COG and stimulator output intensity was set to the intensity of the inflection point of the recruitment curve. After the final PAM mapping, two recruitment curves were recorded at the COG of the second PAM mapping; the first during pre-innervation and the second with the muscle at rest.

### Data analysis

Data analysis was done with Matlab (MathWorks, Natick, MA). The MEP peak-to-peak amplitudes of each grid point served for the construction of the topographic maps and COGs (Wassermann et al., 1992). The maps were created using relative values for the amplitudes according to the maximum and minimum peak-to-peak amplitude on a stimulated grid point. As the FDI mapping was performed with the muscle in rest, the baseline level was near 0 μV. Because of pre-innervation during PAM mapping, a cut-off was introduced to increase the ratio between the MEPs signal and pre-innervation activity. For this, the lowest and highest MEP amplitudes were chosen from the entire target grid (Vmin and Vmax). At each target the lowest MEP amplitude and half of the difference between lowest and highest MEP amplitude were subtracted from the mean MEP amplitude (Vi) of the respective target:

$$v_{i^{*}}=v_{i}-v_{\min}-\left(v_{\max}-v_{\min}\right)/2$$

From the data of all *v*_i*_>0 the COGs were calculated using the following formula:

$$COG=\sum v_{i^{*}}x_{i}/\sum v_{i^{*}},\;\sum v_{i^{*}}y_{i}/\sum v_{i^{*}};\;for\;all\;v_{i^{*}}>0$$

For the neuroanatomical positioning of the representational area on the brain surface, the individual grid points and COGs were projected onto the dura mater by extrapolating the vector that was used to maintain a constant angle of the coil during the experiments (along the stimulation axis of the coil’s magnetic field through the center of the TMS coil). This vector was perpendicular to the tangent of the scalp surface at the stimulated grid point. COGs were displayed on a co-registered individual brain surface within the MNI152 T1 1 mm brain template using FSL (BET, FNIRT and FLIRT).

### Functional magnetic resonance imaging

The subjects were placed supine in a Magnetom TIM Trio scanner (Siemens Healthcare, Erlangen, Germany) operating at 3 T. A standard 8-channel phased-array head coil was used. For noise protection subjects wore earplugs. For each subject two scan runs were performed in one session. Initially, T1-weighted anatomical images of the head at 1mm^3^ isotropic resolution were acquired for each participant (3D turbo FLASH, repetition time (TR): 2250 ms, inversion time: 900 ms, echo time (TE): 3.26 ms, and flip angle: 9°).

For functional BOLD imaging a T2*-sensitive gradient-echo EPI technique with an in plane resolution of 2 × 2 mm^2^ was used (TR: 2000 ms, TE: 36 ms, flip angle: 70°, acquisition matrix: 128 × 96). 22 consecutive sections 3 of mm thickness angulated in axial-to-coronal orientation (covering the primary-, pre- and somatosensory cortex as well as the supplementary motor area) were acquired. For each of the two functional runs 129 volumes were recorded. Subjects were cued by a red or green disk to either rest or perform movement in a blocked design with eight 12-second blocks of movement separated by nine 18-second blocks of rest. In each run one type of movement was executed, either index finger movement or PAM activation. For the index finger motor task, subjects were instructed to move both index fingers from side to side at a frequency of about 0.5 Hz. For the ear muscle motor task, subjects were instructed to perform the same motor task as used for the TMS experiments. The motor task consisted of repeatedly moving the pinna backwards as strongly as possible. Subjects were instructed to hold the activation for a second without activating other facial muscles or moving the head. However, two subjects repeated the scanning session because of substantial task-correlated head motion during PAM activation in the first session.

BrainVoyager QX (Brain Innovation, Maastricht, The Netherlands), Matlab and the NeuroElf toolbox (http://neuroelf.net) were utilized for image preprocessing and statistical analysis. Preprocessing included slice scan time correction, motion correction and temporal high pass filtering using standard parameters (a general linear model with a Fourier basis set consisting of sines and cosines up to 2 cycles/run and a linear trend predictor). Subsequently, functional datasets were co-registered onto the T1-anatomical datasets of the respective subject, transformed to Talairach space and smoothed with a Gaussian filter of 5 mm full-width at half-maximum.

The general linear model was used to analyze the fMRI data. Boxcar predictors for movement blocks were convolved with the canonical two gamma hemodynamic response function to generate predictors of movement related activity. In addition, the six movement parameters from the motion correction were included as regressors of no interest.

For whole brain group analysis the random effects model served to illustrate the main effects (PAM vs. rest, and FDI vs. rest). This model allows a generalization of the results to the population. For thresholding of the whole brain random effects group maps (RFX maps) the false discovery rate (FDR) q < 0.05 (corresponding to t(12) > 4.30 for PAM, and t(12) > 3.65 for FDI) was used. As the corresponding t-thresholds differed between the two movements, we adopted a common threshold of p(uncorr.) < 0.001 (t(12) > 4.32) for both maps. This is slightly stricter then the less liberal FDR q < 0.05 threshold for the PAM main effects.

To analyze group activation in the primary motor cortex, the biggest clusters of fMRI activity in the primary motor cortex for both movements were isolated in each subject. Individual patches of interest for the primary motor cortex were defined on the cortical surface representation of each subject’s individual anatomy using the depth of the central sulcus as the posterior, the crown of the precentral gyrus as the anterior, the superior convexity as the medial and the lateral sulcus as the lateral border. This primary motor cortex representation was projected back to Talairach space to yield individual regions of interest (ROI) for the primary motor cortex. Individual activation maps (clusters of activity) for movement of the pinna and index finger were generated and the threshold was set at t(128) > 5.00. Then all clusters of activity in the primary motor cortex were imported through the NeuroElf Toolbox to Matlab for further analysis. A cluster size threshold of 50 mm^3^ was applied to increase reliability. The largest cluster was selected as the most likely center of primary motor cortex activity for the movement and COG coordinates were calculated from this cluster (using voxel t-values as weights). These most relevant clusters in the primary motor cortex and their COGs were visualized within the Talairach space. For group analysis the mean COG was calculated from the individual COG coordinates.

## Results

### Motor evoked potentials

**Fig 3:**
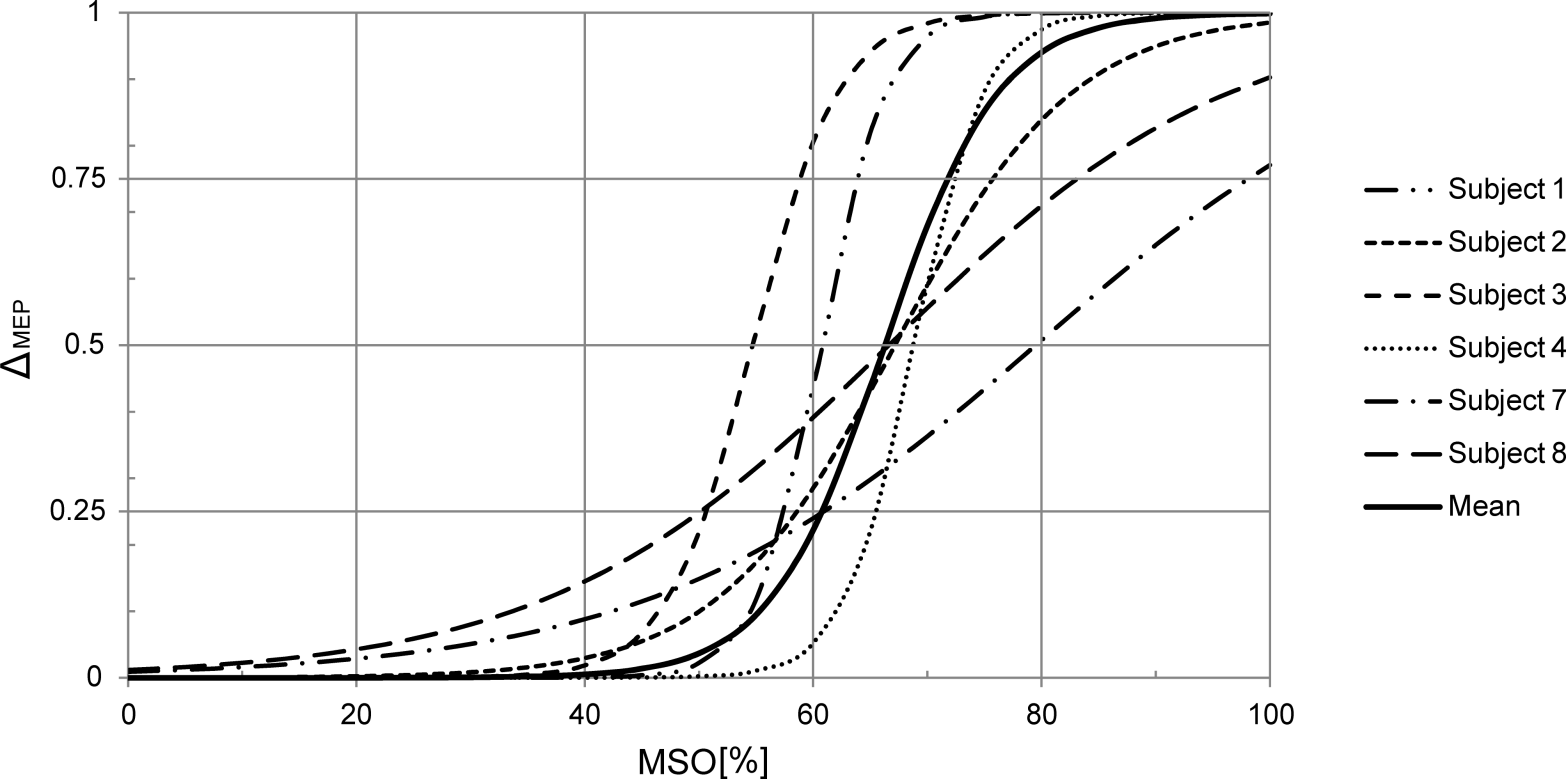
TMS recruitment curves. TMS recruitment curves over the COGs of PAM modeled as sigmoid functions. The resulting MEPs were fitted to the sigmoid function f(x) = *amp*_*nostim*_+(*amp*_*maxstim*_*—amp*_*nostim*_)/(1+exp(m*(W-x))). *amp*_*nostim*_ is the lower amplitude level consisting only of pre-innervation activity, *amp*_*maxstim*_ is the upper amplitude level equivalent to the maximum MEP amplitude at supramaximal stimulation. M is the steepness of the sigmoid curve at the point of inflection W. Individual differences of the MEPs in the subjects are ignored by setting *amp*_*nostim*_ = 0 and *amp*_*maxstim*_ = 1, so the function is simplified to Δ_MEP_ = 1/(1+exp(m*(W-x)).

Pre-innervation EMG amplitudes ranged from 100 to 509 μV (mean: 168 μV; SD: 59) in subject 2 (lowest pre-innervation level reached) and 270 and 3900 μV (mean: 1700 μV; SD: 510) in subject 4 (highest pre-innervation level reached). MEPs from PAM were consistently recorded in all subjects. MEP amplitudes depended on the stimulation intensity (Fig 3). The relative values for amplitudes and the stimulator output intensity were fitted to a sigmoid function. The point of inflection was between 55% and 79% of MSO in the majority of the subjects (6 out of 8) (Fig 3). In the remaining two subjects no supra-maximal stimulation was achieved.

**Fig 4:**
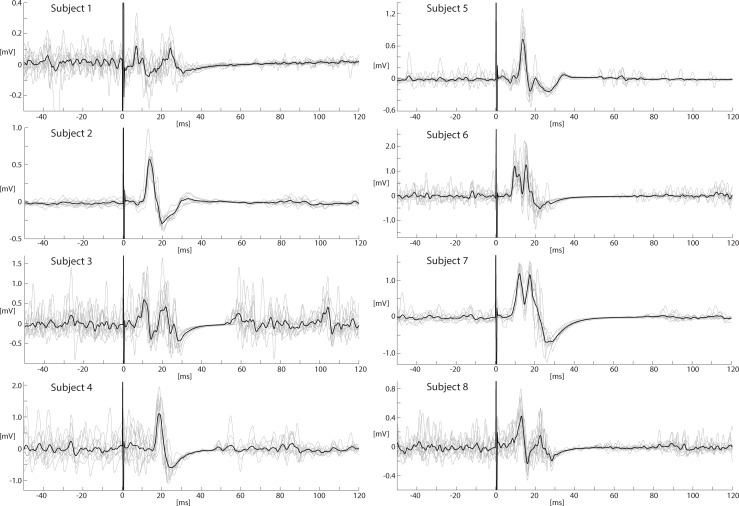
MEPs from the PAM. Individual MEPs (gray lines) and averages (black line) recorded from the pre-activated PAM over the COG of subjects 1–8 at the stimulation intensity next to the inflection point of the recruitment curves + 10% (subject 1: 70%, subject 2: 80%, subject 3: 60%, subject 4: 80%, subject 7: 80%, subject 8: 80% of maximum stimulator output). In the subjects where no inflection point was observed (subject 5 and 6), the MEPs from the maximum intensity were used. The mean onset latency L0 was 9.2 ms (SD: 0.9 ms), the mean peak latency LP 12.6 ms (SD: 1.1 ms).

The shape of the MEPs was variable across the subjects. While in most subjects (5 out of 8) a second major peak with inconsistent amplitude and latency could be observed, some subjects (3 out of 8) had a single peak MEP conformation (Fig 4). Higher stimulation intensities tended to result in a single peak confirmation. The mean onset latency was 9.2 ms (SD: 0.9) and the mean (first) peak latency 12.6 ms (SD: 1.1) at the intensity of stimulator output next to the inflection point of the recruitment curves (Fig 4). Recruitment curves obtained with the muscle at rest were recorded in 5 out of 8 subjects. MEPs from the resting muscle showed a single peak configuration and a positive recruitment. Compared to the pre-activated muscle the MEP amplitudes higher stimulation intensities were required to elicit MEPs. In the noise artifact control experiment none of the subjects showed a PAMR.

### Cortical TMS representation of the posterior auricular muscle and the first dorsal interosseus muscle

**Fig 5:**
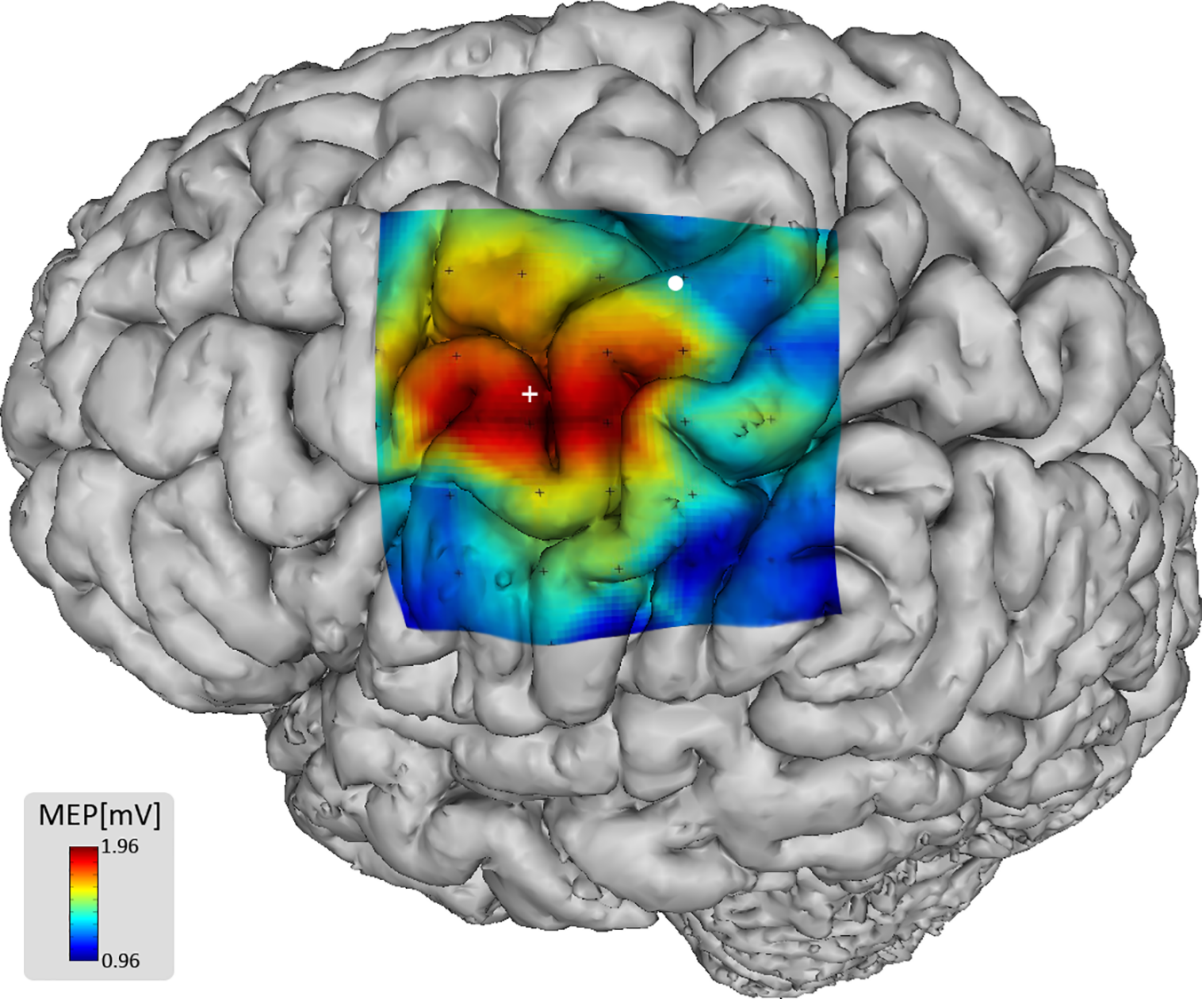
Projection of the individual PAM TMS mapping results onto the cortical surface of subject 3. The Projection of stimulation sites (grid points, black crosses), PAM COG (white cross), MEP amplitudes (colors) and FDI COG (white dot) was determined by extrapolating the z-vector of the TMS coil, which is orthogonal to its plane, onto the cortical surface.

**Fig 6:**
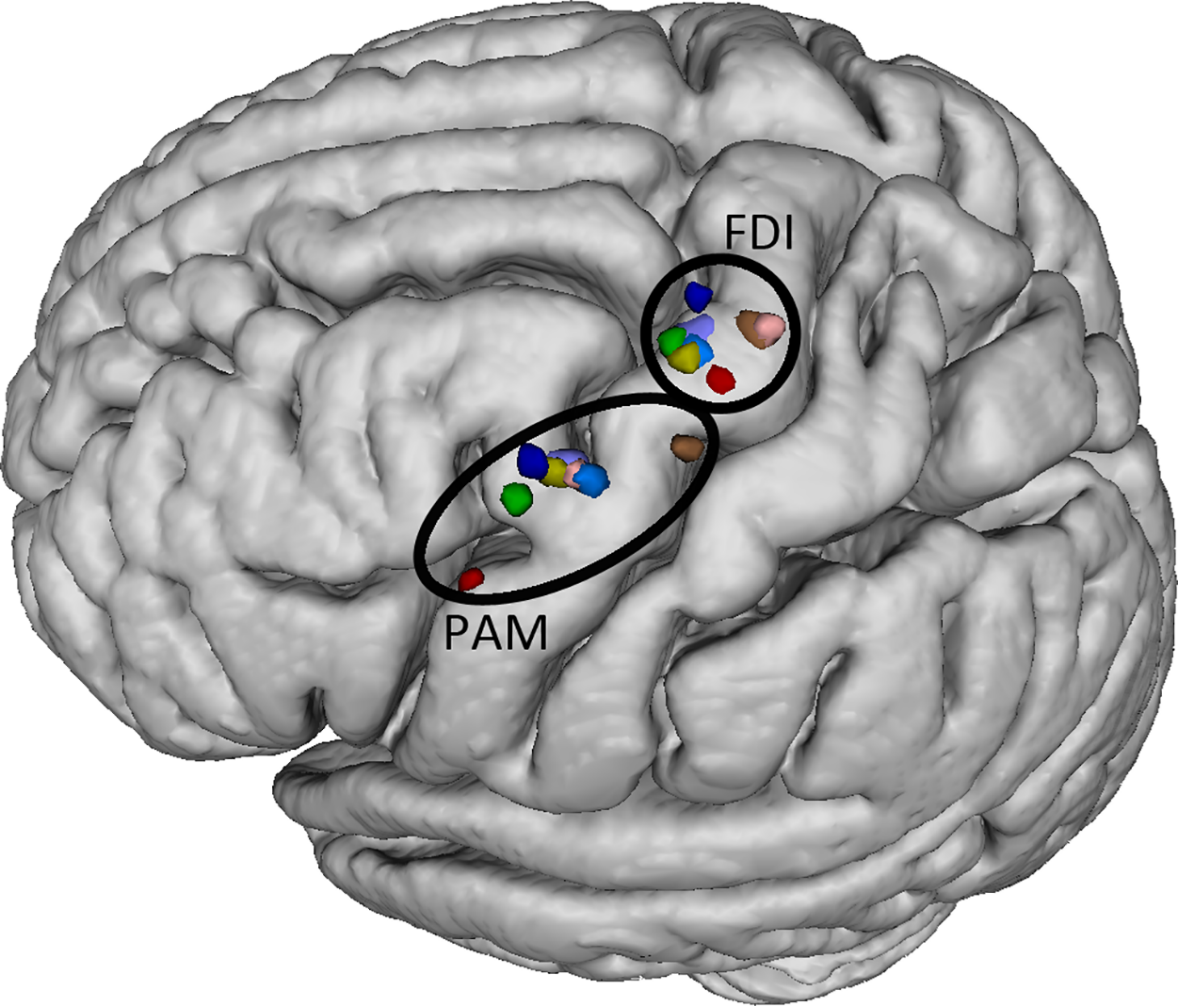
Positions of individual COGs of FDI and PAM on the MNI152 T1 template. Colors indicate different subjects. The average distance between the individual PAM and FDI COGs is 23.8 mm (SD: 5.1 mm). The individual TMS PAM coordinates were [-57, 9, 39] in subject 1; [-48, 4, 52] in subject 2; [-50, -1, 53] in subject 3; [-50, 7, 48] in subject 4; [-50, 0, 53] in subject 5; [-49, -10, 60] in subject 6; [-49, 6, 53] in subject 7 and [-47, 2, 51] in subject 8.

**Fig 7:**
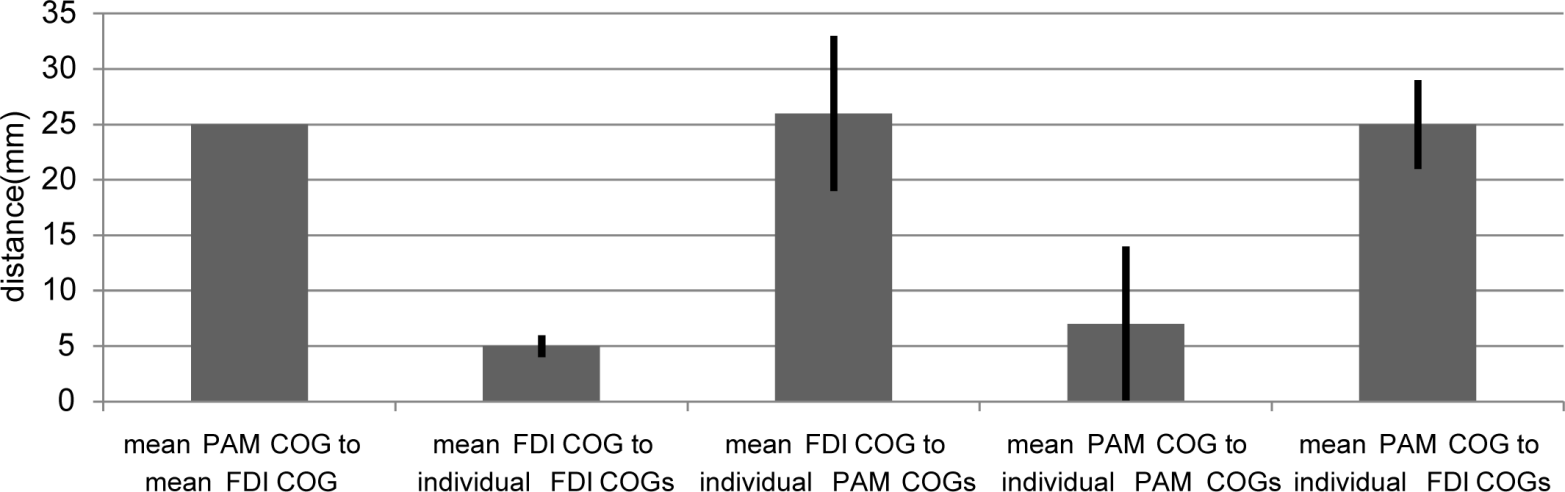
Distances between PAM and FDI COGs. Distances (in mm) between mean and individual PAM and FDI COGs calculated from TMS mapping. Error bars indicate standard deviation.

In the FDI experiment, MEPs were elicited when stimulating a circumscribed area of the precentral gyrus that included the hand knob. This area was round in shape. The COGs were located within the precentral gyrus on a level with the medial frontal gyrus. In 5 out of 8 subjects the COGs were positioned in the anterior part of the gyrus, close to the precentral sulcus. In the remaining subjects the COGs were located more posteriorly, in the middle of the precentral gyrus (Figs 5 and 6). Mean coordinates of the COG positions in the MNI152 space were [-36, -12 and 67]. The dispersion of the COGs, i.e. the distance between the individual and the mean COGs, was 5 mm (SD: 1) (Figs 6 and 7).

MEP amplitudes in the PAM experiment were elicited with the TMS coil placed over a defined area including parts of the precentral gyrus. The oval-shaped PAM area was located several millimeters lateral to the hand knob. In 6 out of 8 subjects the COGs were consistently positioned in a confined area within the precentral gyrus on a level with lower parts of the medial frontal gyrus (Figs 5 and 6). In the remaining two subjects the COGs were located more distant on the precentral gyrus; in one subject more anterolaterally (next to the precentral sulcus, on a level with the inferior frontal gyrus) and in the other subject more posteromedially (next to the central sulcus) (Figs 5 and 6). Mean coordinates in the MNI152 space were [-50, 2 and 51]. The distance between the individual COGs of the PAM and its mean COG was 7 mm (SD: 5) (Figs 6 and 7).

On the level of the cortical surface, the mean distance between the individual PAM and FDI COGs was 23.8 mm (SD: 5.1). The distance between the mean COGs of both muscles was 26.3 mm.

### Functional magnetic resonance imaging

**Fig 8:**
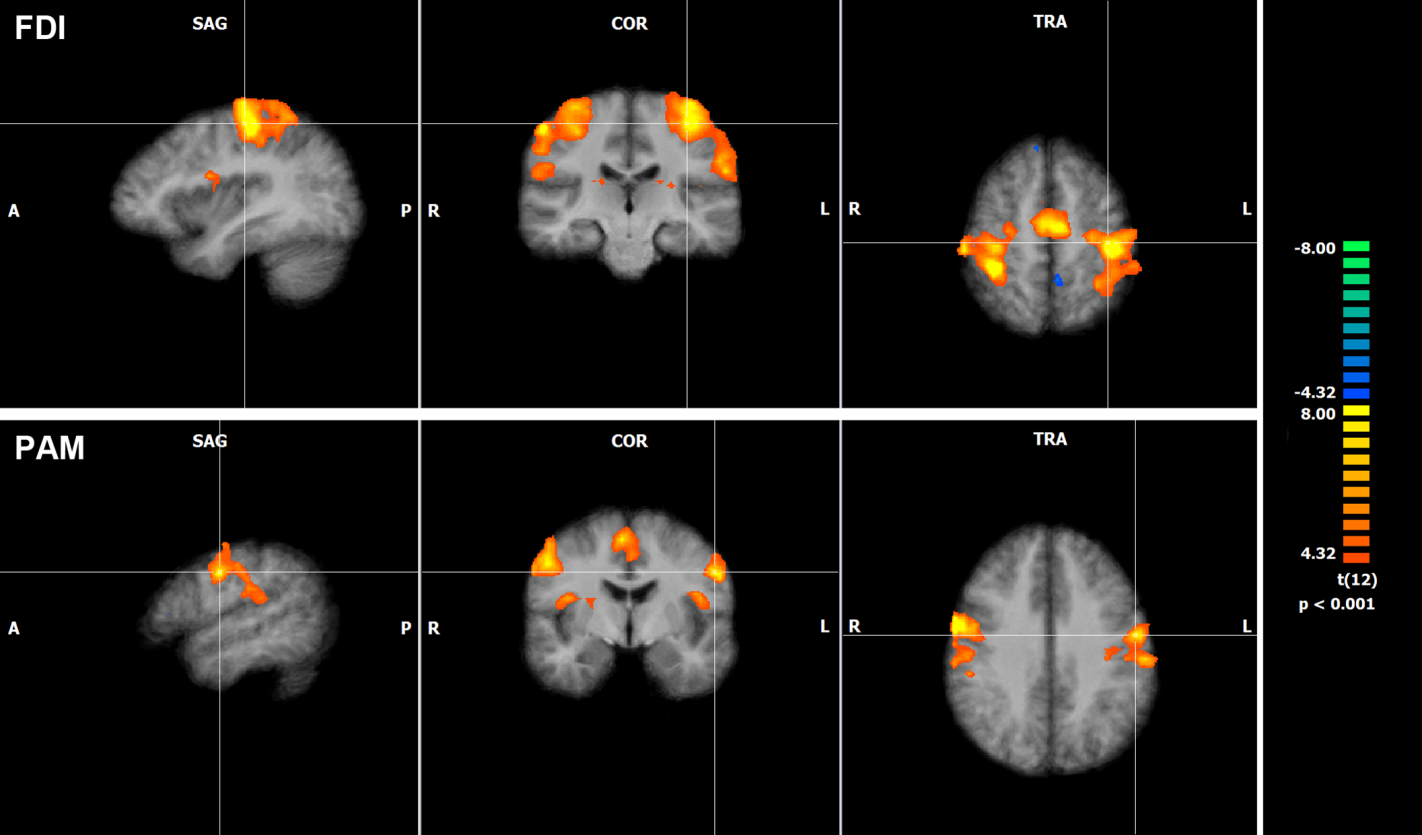
fMRI COGs of PAM and FDI activation. fMRI group activation maps for PAM and FDI with crosses through the mean COGs in a sagittal, coronar and transversal plane (P < 0.001).

**Fig 9:**
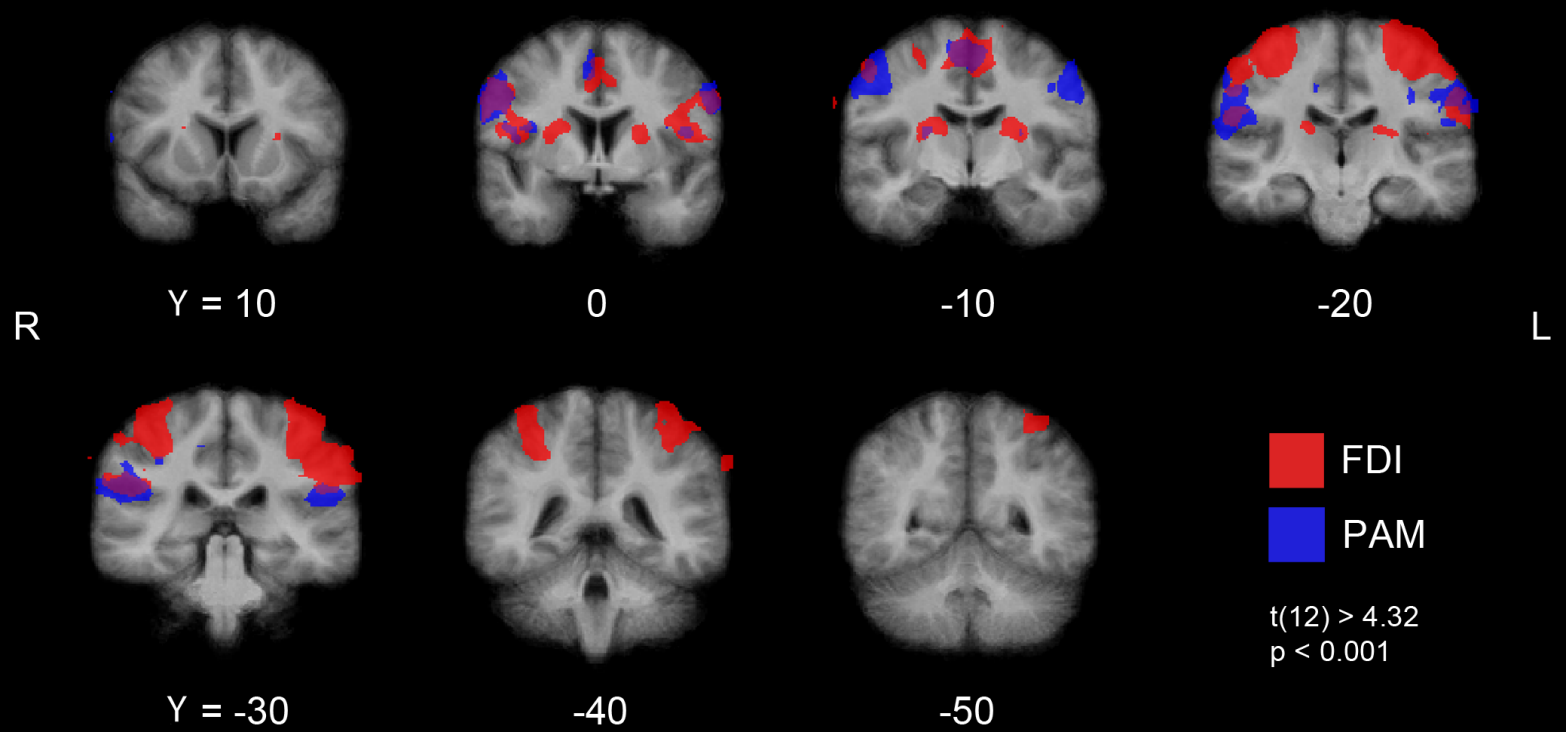
Cortical fMRI activation during PAM and FDI activation. Series of coronar fMRI images at Talairach Y = 10, 0, -10, -20, -30, -40, -50 with cortical activation during index finger movement (red), movement of the pinna (blue) and overlapping areas (purple) (P < 0.001; t(12) > 4.32).

In the group analysis, finger tapping and movement of the pinna resulted in bilateral patterns of increased BOLD signal as compared to rest. The signal to the index finger motor task was stronger and more widespread than to the movement of the pinna. During movement of the pinna, increased BOLD signal was recorded bilaterally within the primary motor cortex, somatosensory cortex, supplementary motor area (SMA), insula and basal ganglia. Index finger movement related increased BOLD signal was detected bilaterally within the primary motor cortex, somatosensory cortex extending into parietal areas, premotor cortex, SMA, basal ganglia and insula (Figs 8 and 9).

Thalamus and deeper brain structures were not covered by the fMRI slice package. For single subject analysis, in two of the subjects clusters of activity for movement of the pinna were absent at the chosen threshold in the left primary motor cortex and in another subject in the right primary motor cortex. These subjects were excluded from further analysis of the ROI (anatomically defined primary motor cortex) of the corresponding hemisphere.

**Fig 10:**
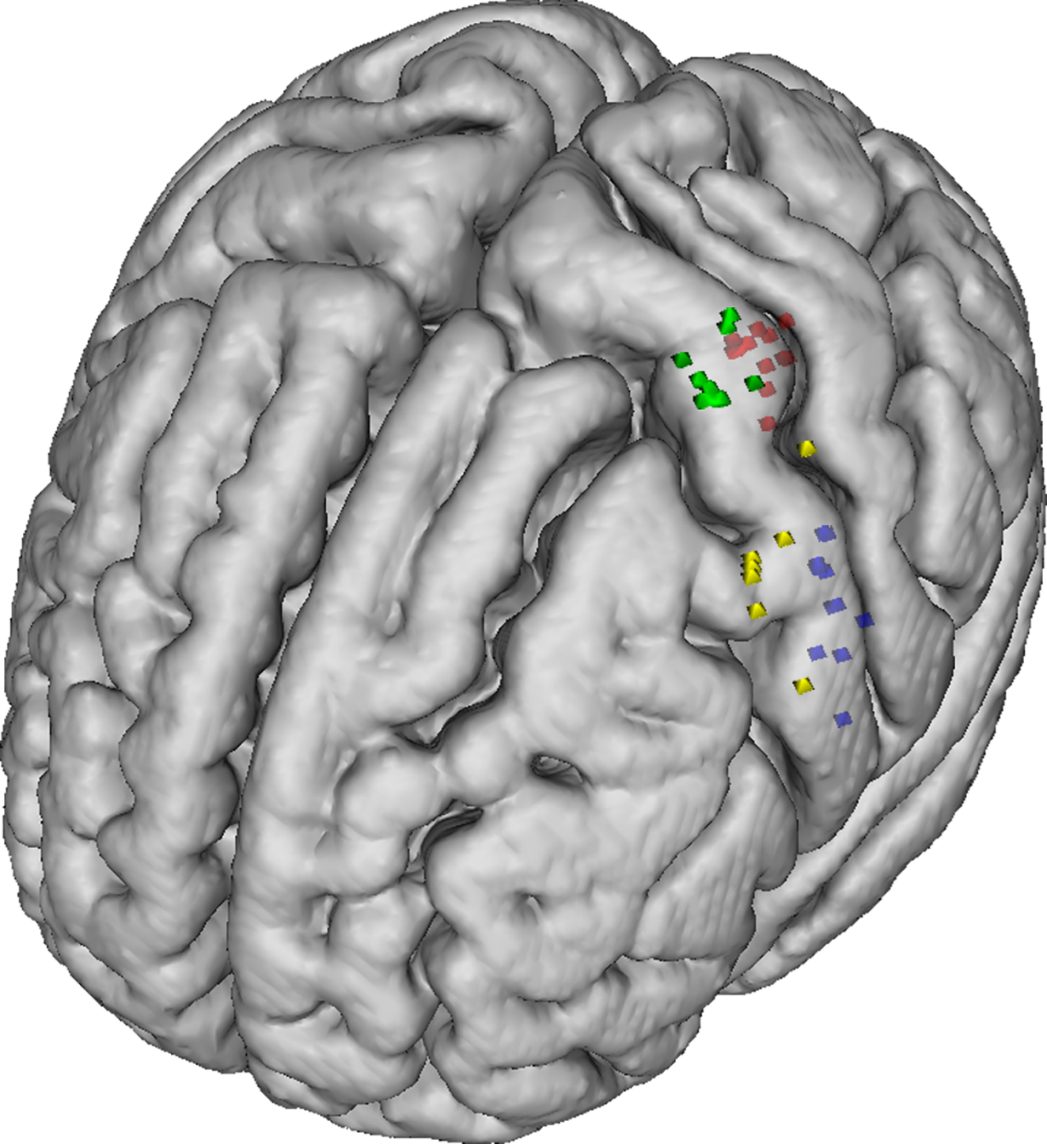
Positions of the individual fMRI and TMS COGs of PAM and FDI registered to the MNI152 T1 template. The TMS PAM COGs (yellow) are located on the precentral gyrus lateral to the TMS FDI COGs (green). The fMRI PAM (blue) and FDI (red) COGs are located more posterior on the precentral gyrus in comparison to the TMS COGs.

In the ROI, the mean cluster size of the left and right hemispheric representation of PAM was 1154 mm^3^ and 1032 mm^3^. The mean FDI cluster size covered 2590 mm^3^ in the left and 3077 mm^3^ in the right hemisphere. The coordinates of mean activation COGs during the respective motor tasks on the left and right hemisphere were the same but mirror-inverted. On the precentral gyrus, the individual and mean PAM COGs were arranged more inferiorly, laterally and anteriorly in comparison to the FDI COGs (Figs 8, 9 and 10).

In both hemispheres, the dispersion of the individual COGs, i.e. the distance between the individual COGs and the mean COGs, was larger for the movement of the pinna than for the index finger. Mean COGs of the activation area during both motor tasks were located distinct of each other. FDI COGs were located on the precentral gyrus close to the anterior bank of the central sulcus on a level with the medial frontal gyrus. PAM COGs were located on the precentral gyrus close to the central sulcus and on a level with the inferior frontal gyrus.

In addition, the increased activation extended from the precentral gyrus into neighboring postcentral and inferior parietal cortex.

## Discussion

Using a novel automated robotic high-precision TMS procedure and fMRI we identified, for the first time, the cortical representation of the auricular muscles in humans. The auricular muscle representation area was located within the primary motor cortex lateral to the hand area. The results were consistent across subjects. We further achieved good correspondence between TMS and fMRI. Finally, our results confirm that all subjects are able to generate PAM EMG signals on request.

### Motor cortical representation of the auricular muscles in humans

The auricular muscle representation area identified with TMS and fMRI was located predominantly within the primary motor cortex lateral to the FDI area. A similar cortical representation has previously been identified for different muscles of the lower face with TMS and fMRI [27–33]. The cortical representation of upper facial muscles is, in contrast, still a subject of debate. While studies in non-human primates revealed only sparse projections from the primary motor cortex to the upper facial muscles [9, 10], studies in humans suggest a representation within the primary motor cortex [29, 30, 32]. The MEPs are, however, less reproducible in upper facial muscles compared to lower facial muscles [32]. Besides being less represented it has been discussed that upper facial muscles might have a higher stimulation threshold [34]. The present study was the first to investigate the cortical representation of the PAM in humans. The results, i. e. the identified representation area within the primary motor cortex, are interesting for several reasons. Firstly, our results indicate that the cortical representation of auricular muscles in humans differs from non-human primates where the auricular muscles are mainly represented within supplementary motor areas rather than within the primary motor cortex where most of the lower facial and probably also the upper facial muscles are represented [9, 10, 29, 32]. This difference points to an evolved function of the auricular muscles in humans. While in humans apart from its integration in reflex activation as in case of PAMR [4, 35, 36] the function of the auricular muscles is unknown, auricular muscles are assumed to play an important role in locating acoustical sources in non-human species. Charles Darwin observed, that some animals had flabby ears and proposed that the ear muscle function (for the location of acoustical sources) might have disappeared in some species during domestication [7]. However, considering our mapping results and the observation that all subjects were able to activate the PAM on request a function in humans (beyond its integration in short-latency brainstem reflexes) is likely but still has to be elucidated. An example could be the activation of the auricular muscles during different facial expressions [37].

Secondly, our results point to a cortical origin of auricular muscle activation in humans. We therefore assume that if auricular muscle activation was used as a human machine interface e. g. for wheelchair control, humans should be able to adept and learn to activate the auricular muscles in this context [8]. The idea behind using auricular muscle activation as a control signal for human machine interfaces is twofold. As the original function of the auricular muscles in humans is probably negligible, a PAM based control system should not interfere with any motor function if used as a human machine interface. This is in contrast to human machine interfaces based on hand, chin or tongue movements for example [8]. Moreover, it would be also suitable for tetraplegics, i. e. patients without remaining motor function of the arms and hands.

Thirdly, the PAM and FDI areas are located relatively close to each other. This neuroanatomical vicinity of both muscle representation areas could be of advantage for patients with tetraplegia. We hypothesize that the orphaned hand motor cortex could be recruited by the adjacent auricular muscle cortex if it was used to control a wheelchair for example. This hypothesis is supported by studies on cortical plasticity and reorganization processes in humans. In blind subjects for instance the secondary somatosensory is rerouted to occipital cortical regions [38]. Also peripheral nerve lesions lead to the expansion of close motor representations into the respective non-active area [39–41]. Such a cortical reorganization can occur in either direction between the hand area and representation of facial muscles [41–44] in both the somatosensory cortex and motor cortex probably due to their neuroanatomical vicinity of both cortical areas.

### Motor evoked potentials in the posterior auricular muscle

In all subjects MEPs were recorded in the PAM. The MEP amplitudes and the length of the silent period positively correlated with the stimulation intensity. The shape of the MEPs was different across subjects and depended on the intensity of stimulator output. At lower stimulation intensities in most subjects a two peak conformation was observed. At the intensity of the inflection point + 10%, 5 out of 8 subjects showed a two-peak confirmation and 3 out of 8 had a single peak MEP conformation. The two-peak conformation disappeared in most of the subjects at higher stimulation intensities. The MEP is a compound potential, i. e. a summation of potentials of different motor units. The two-peak conformation might therefore result from three factors; first, different nerve conduction times of the stimulated motor neurons; second, a relatively low number of (stimulated) motor neurons and third, a high stimulation threshold.

A drawback of somatotopic TMS studies in general is a possible contamination of the MEPs by cross talk from adjacent muscles. However, for several reasons it is unlikely that the PAM MEPs were volume conducted potentials from distant muscles. We used fine-wire electrodes and a special insertion technique for the experiment. In the middle of the electrode the insulation was removed at approx. 1 cm. The electrode was positioned in a loop through the muscle with the non-insulated part positioned subcutaneously, i. e. within the muscle. Cross talk from distant muscles was therefore avoided. Another observation that argues against the measurement of volume conducted potentials, is the mean onset latency of PAM MEPs. While it was 9.2 ms (SD: 0.9) in the PAM latencies between 10 and 14 ms have been reported for other upper and lower facial muscles [29, 32]. The shorter latency of the PAM MEPs probably results from the shorter length of the first branch of the facial nerve that is connected with the PAM as compared to other branches of the facial nerve. MEPs of the tongue muscle, however, have a latency in a similar range as the PAM [45], but as the PAM is located distant to the tongue muscle behind the pinna and since fine wire electrodes were used for the EMG recording a major impact on the PAM MEPs by volume conducted potentials from the tongue is unlikely. Moreover, the cortical representation of the tongue is supposed to be located more lateral compared to the identified PAM representation area [30].

Besides volume conduction the PAMR is another possible confounder of PAM MEPs. However, a contamination of PAM MEPs by the PAMR is unlikely for four reasons. Firstly, none of the subjects showed a PAMR in the control experiment. Although the control experiment has the limitation that the center of the coil was not placed directly on the scalp resulting in a less loud noise artifact a strong PAMR is improbable. Secondly, a TMS pulse would have triggered the PAMR independently at each grid point. The absence of MEPs when stimulating the edge of the grid therefore argues for a true cortical origin of the TMS-evoked responses. Thirdly, the identification of a corresponding cortical representation area of the auricular muscles using fMRI further confirms that the measured MEPs originate in the primary motor cortex and were not caused by reflex activation. Fourthly, a silent period was observed in all subjects. The length of the silent period was approx. in a similar range or somewhat shorter as reported for other facial muscles [29, 46] and depended on the stimulation intensity. The longest silent periods were observed at targets close to the COG. This observation also points to a true cortical origin of the silent period. An inhibition caused by the trigemino-facial inhibitory reflex that can be observed in some facial muscles at a similar latency after trigeminal stimulation [47] is therefore unlikely.

### Transcranial magnetic stimulation mapping and functional magnetic resonance imaging

To confirm the TMS mapping results in a cross-modal approach and to possibly identify neuronal activation during auricular muscle activation outside primary motor cortex, we performed an additional fMRI mapping. Both, movement of the index finger and movement of the pinna resulted in an increased BOLD signal within the primary motor cortex, somatosensory cortex, SMA, insula and basal ganglia. Additionally, the premotor area was also activated during the index finger motor task. The activation during the PAM motor task was located more lateral on the precentral gyrus compared to the index finger motor task. A previous fMRI study found a similar somatotopy within the primary motor cortex during movement of the hand and lips [30]. In our study the fMRI activation patterns during the index finger motor task were more pronounced compared to activation during movement of the pinna. This observation is in accordance with previous fMRI studies that showed a stronger activation during finger motor tasks compared to movements of tongue, lips, arms or legs [28, 30]. One possible explanation of this observation is that finger movement requires a very fine motor control, which includes the activation of different hand and arm muscles. It is therefore very likely that further muscles have been involved during the FDI motor task resulting in a stronger fMRI activation.

The TMS experiments revealed opposite results with the PAM area appearing more widespread than the FDI area. Movement of facial muscles is supposed to be controlled by a large, somatotopic organized neuronal network composed by overlapping brain areas [30, 48]. The PAM representation area might be part of such a network resulting in a more widespread appearance of the TMS PAM area compared to the TMS FDI area. However, besides neuroanatomical differences three methodic factors, that are known to influence TMS results [16, 49, 50], might have contributed to this observation as well. Firstly, the stimulation intensity was considerably higher for the PAM experiment compared to the FDI experiment. Secondly, the PAM experiments were conducted during pre-innervation. It has been shown previously that both factors influence the size of the area on the scalp where MEPs are elicited with TMS [50]. Thirdly, the rotation angle was set to 45°, that has been proven suitable for hand muscle TMS [51]. As this was the first time the PAM was investigated with TMS, it is possible that the optimal coil rotation angle for the PAM is different. An optimized rotation angle would have probably resulted in a more confined PAM area. The influence of the coil orientation [52], stimulation intensity and pre-activation of the muscle [50] on the COG location is, in contrast, minimal. The different TMS protocols used for the PAM and FDI experiments should therefore not have influenced the results (COG location).

The COGs of PAM and FDI were both located on the precentral gyus but significantly distant from each other. The dispersion of the individual COGs, i.e. the distance between the individual and the mean COGs, was higher for the PAM than for the FDI. This might be due to an increased inter-subject variability with respect to the exact cortical representation of the PAM or a less focused location of the corresponding neurons in comparison to the FDI.

The TMS COGs were located somewhat more anterior as compared to the fMRI COGs. This shift is consistent with previous studies that compared TMS mapping with fMRI [11, 53, 54] and might be the result of several methodical differences of both mapping techniques. While fMRI measures an increased blood-oxygen-level during cortical activation, TMS induces a transmembrane current that stimulates neuronal cells, which is thought to happen preferentially at ends or bends of axons [55–57]. As a result, the location of the intracortical stimulation does not necessarily correspond to the increased blood-oxygen-level that is caused by a higher metabolism of oxygen within the neuronal cell bodies. Furthermore, the current flow is shaped by the brain tissue [58]. As a results the stimulation site not necessarily corresponds to the location where the current is highest [58, 59]. The current flow that finally leads to the neural depolarization is therefore not necessarily located precisely under the center of the TMS coil [56]. This could then result in a discrepancy between the expected stimulation site (under the center of the coil) and the real position of the stimulated neurons within the cortex. Another contributing factor might be the lack of muscle specificity during fMRI mapping. Although the subjects were instructed to activate mainly the PAM and FDI, it is likely that other muscles have been involved during the motor tasks and it was not controlled inside the scanner if the motor task was performed correctly. This might have also influenced the location of the fMRI COG.
